# Schistosomiasis in the Military—A Narrative Review

**DOI:** 10.3390/tropicalmed9090221

**Published:** 2024-09-19

**Authors:** Diana Isabela Costescu Strachinaru, Jemima Nyaboke Nyandwaro, Anke Stoefs, Eric Dooms, Peter Vanbrabant, Pierre-Michel François, Mihai Strachinaru, Marjan Van Esbroeck, Emmanuel Bottieau, Patrick Soentjens

**Affiliations:** 1 Center for Infectious Diseases, Queen Astrid Military Hospital, 1120 Brussels, Belgium; eric.dooms@mil.be (E.D.); peter.vanbrabant@mil.be (P.V.); psoentjens@itg.be (P.S.); 2 Department of Clinical Sciences, Institute of Tropical Medicine, 2000 Antwerp, Belgium; nyabokejemima@gmail.com (J.N.N.); mvesbroeck@itg.be (M.V.E.); ebottieau@itg.be (E.B.); 3 Department of Microbiology, Queen Astrid Military Hospital, 1120 Brussels, Belgium; anke.stoefs@mil.be; 4 Medical Component Operational Command, Queen Astrid Military Hospital, 1120 Brussels, Belgium; pierre-michel.francois@mil.be; 5 Department of Cardiology, Brussels University Hospital—Erasme Hospital, Université Libre de Bruxelles, 1070 Brussels, Belgium; mihai.strachinaru@hubruxelles.be

**Keywords:** schistosomiasis, *Schistosoma* spp., neglected tropical disease, imported disease, military, veterans, tropical deployment, tropical medicine, travel medicine

## Abstract

Schistosomiasis is a parasitosis caused by trematodes of the genus *Schistosoma*. Humans are infected when coming into contact with freshwater containing the parasites’ infective stages, which are amplified through freshwater-dwelling snails acting as intermediate hosts. Schistosomiasis has posed significant problems for troops exposed to freshwater in endemic regions ever since the Napoleonic wars. Schistosomiasis has substantial differences in clinical presentation, depending on the type of parasite, intensity of infection and reinfection, clinical form, and disease stage. It can remain undiagnosed for long periods of time, with well-known long-term morbidity and mortality risks. The diagnosis of schistosomiasis depends on its stage and relays on several tests, all with limitations in sensitivity and specificity. The diagnostic gold standard is the detection of eggs in urine, feces, or tissue biopsies, but this can raise problems in patients such as military personnel, in which the worm burden is usually low. Praziquantel is the drug of choice for schistosomiasis. Currently, there is no available commercial vaccine against any *Schistosoma* parasite. Avoiding freshwater exposure is the best prevention. Herein, we review the clinical presentation, diagnosis, treatment, and prevention of schistosomiasis in the military. This information may decrease the impact of schistosomiasis on this particular professional group.

## 1. Introduction

Schistosomiasis is a parasitic disease caused by trematode worms (flukes) of the genus *Schistosoma*, whose infective stages, the cercariae, are amplified through freshwater-dwelling mollusks (snails) acting as intermediate hosts [1,2]. Humans are infected when coming in contact with freshwater containing cercariae that penetrate through the skin [1,2]. Schistosomiasis is a common parasitic disease which is prevalent in tropical and subtropical areas, especially in poor communities without access to safe drinking water and adequate sanitation [1,3]. Transmission has been reported from 78 countries, but it is estimated that more than 90% of those requiring treatment for schistosomiasis live in Africa [1,4,5]. There are two major forms of schistosomiasis, intestinal and urogenital, caused by five main species of blood flukes: *Schistosoma haematobium* (which causes urogenital schistosomiasis), *Schistosoma mansoni*, *Schistosoma japonicum*, *Schistosoma mekongi*, and *Schistosoma guineensis* (and the close-related *Schistosoma intercalatum*), which cause intestinal and hepatosplenic schistosomiasis [1,5]. The species responsible for most human infections are *S. haematobium* and *S. mansoni* [1,2]. The clinical spectrum of schistosomiasis is very varied, depending on the pattern of exposure, the intensity and the stage of infection, and the infective species [4,5]. Schistosomiasis in travelers differs substantially from infection in endemic populations in many aspects, such as level of exposure, morbidity, treatment, and prevention [2,4,5]. In travelers, it may present as an acute or chronic disease, with or without stage-specific typical signs and symptoms [2,3,4,5,6,7]. Military personnel deployed in endemic areas, who are frequently exposed to freshwater through the nature of their jobs, are a unique group of travelers at high risk of schistosomiasis infections [8,9,10,11]. The aim of this study is to provide the medical and nursing personnel caring for active servicemen and veterans with a complete, comprehensible, evidence-based, and up-to-date review of schistosomiasis, including its clinical presentation, current treatment recommendations and available prevention methods, in order to decrease the impact of schistosomiasis on this particular professional group.

## 2. Materials and Methods

This review was conducted in accordance with the Preferred Reporting Items for Systematic Reviews and Meta-Analyses (PRISMA) 2020 checklist [12]. In May 2024, the PubMed database was searched for articles on schistosomiasis in the military, using the following terms: ((schistosomiasis) OR (bilharziasis) OR (schistosoma)) AND ((military) OR (marines) OR (soldiers) OR (armed forces) OR (force health protection) OR (military personnel) OR (veterans)). Based on the inclusion and exclusion criteria listed in Table 1, senior consultant DICS and Master of Sciences student JNN independently screened all retrieved titles and abstracts to identify articles suitable for a full text review.

**Figure 1:**
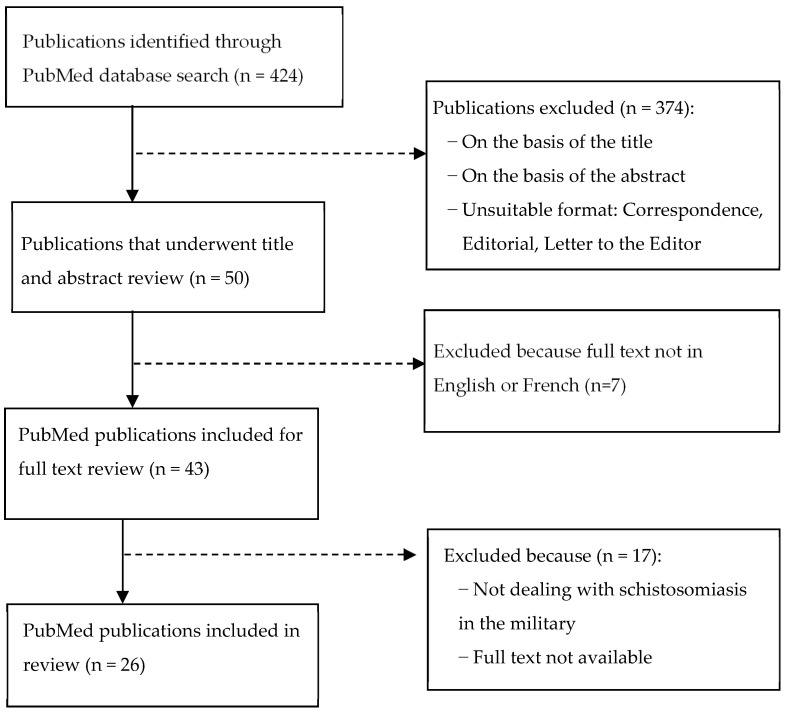
Flowchart showing the selection of publications on schistosomiasis in military personnel.

The results obtained by the two authors were compared and discussed to reach a consensus on which articles should be further analyzed. Next, to find additional references, DICS and JNN examined the bibliographies of the articles that underwent full text review. The study selection process is summarized in Figure 1. Results from articles for which the full text could not be obtained online have not been included.

## 3. Results

Of the 424 articles initially identified after searching the database, 46 were selected by the two authors as eligible for further analysis. After a full reading of the selected articles, 26 met the inclusion criteria and were analyzed in the review synthesis [8,9,10,11,13,14,15,16,17,18,19,20,21,22,23,24,25,26,27,28,29,30,31,32,33,34].

### 3.1. Epidemiology and Life Cycle

**Figure 2:**
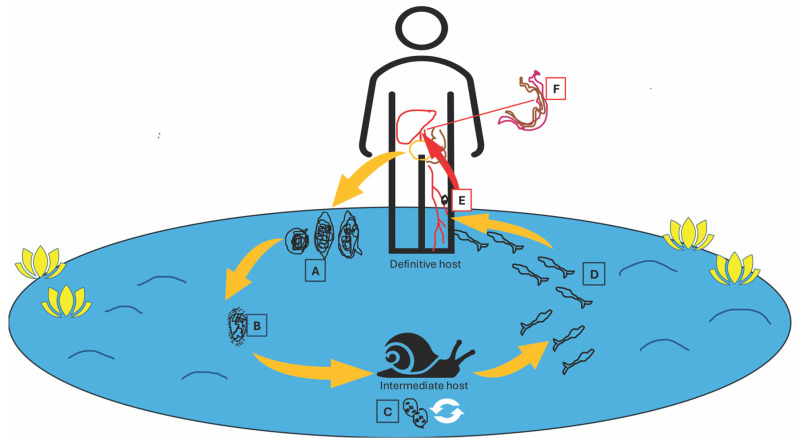
*Schistosoma* life cycle. A: eggs (left to right: *S. japonicum*, *S. haematobium*, *S. mansoni*); B: miracidium; C: sporocysts; D: cercariae; E: schistosomulum; F: a pair of adult worms. 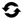: asexual reproduction stage (inside the intermediate hosts).

Schistosomes have a complex life cycle (Figure 2), which involves two consecutive hosts, a freshwater snail and a mammal, and six major developmental stages: eggs, miracidia, sporocysts, cercariae, schistosomula, and adult worms [35]. The asexual reproduction stage occurs in freshwater snails and the sexual stage occurs in mammals [35]. Schistosomiasis was also known as “snail fever”, as the parasites are dependent on their intermediate hosts for reproduction [4]. Each *Schistosoma* species has a limited range of suitable hosts; therefore, their geographic distribution is dependent on that of their respective snails’ habitat range. *S. mansoni* needs certain species of aquatic freshwater *Biomphalaria snails*; snails of the genus *Bulinus* serve as intermediate hosts of *S. haematobium*, *S. intercalatum* and *S. guineensis*, while *S japonicum* needs amphibious freshwater *Oncomelania* snails as its intermediate host [4,35,36]. *S. mekongi* infects snails of the genus *Neutricula* [36]. *S. mansoni* and *S. haematobium* are distributed throughout Africa [1,4]. *S. haematobium* is also found in areas of the Middle East and, in 2014, cases were identified among travelers who had bathed in the Cavu River on Corsica [1,4,37]. *S. mansoni* is also endemic in parts of Brazil, Suriname, Venezuela, and the Caribbean [1,35]. S. japonicum is present in parts of China, in Indonesia, and in the Philippines [1,35]. *S. mekongi* is found in Cambodia and Laos, and *S. intercalatum* in the rainforests of Central and West Africa [1,35].

### 3.2. A Brief History of Schistosomiasis in the Military

Schistosomiasis has been known to pose significant problems for troops exposed to freshwater in tropical environs ever since the Napoleonic wars [9]. During Napoleon’s Egyptian campaigns between 1799 and 1801, French troops suffered severely from hematuria [13]. Theodor Bilharz, a German pathologist, first identified an infective parasite in 1851 while carrying out postmortem examinations on Egyptian soldiers in Cairo: *Distomum haematobium*, renamed *Schistosoma haematobium* by Friedrich Weinland in 1858 [38]. British troops acquired more than 300 schistosomiasis cases during the Boer War [13]. It was a British naval surgeon and parasitologist who studied and helped discover the etiology of schistosomiasis in China before World War I (WWI) [14,15]. Schistosomiasis continued to be a burden for troops deployed in endemic areas during WWI, when several hundred British and Australian soldiers became infected in Egypt and the Middle East [9,13,16]. Even after WWI, schistosomiasis continued having a significant impact on warfare throughout the 20th century [17,39]. During World War II (WWII), schistosomiasis infected troops in several theaters. More than 1500 British and African personnel from the West African Force developed clinical symptoms after exposure in a lagoon in southern Nigeria [9]. German troops studied methods of preventing the infection from 1940 to 1943, while the Afrika Korps were deployed in North Africa [13]. A massive outbreak of *S. japonicum* infections occurred among United States (U.S.) service members during the liberation of the Philippine Islands, when hundreds of troops were infected during the invasion of Leyte [9]. After being exposed to infested waters on Leyte Island, they developed urticaria, cough, abdominal pain, diarrhea, and fever, and often had high eosinophil counts. The diagnosis was formally confirmed when eggs of *S. japonicum* were found in a liver biopsy specimen taken from a soldier who had been evacuated from Leyte [13]. It is also suspected that *S. japonicum* infection was the cause of “Dapeco fever” among U.S. prisoners of war (POWs) and Japanese guards in the Davao penal colony on the island of Mindanao. Many of the symptoms presented by patients with “Dapeco fever” resembled those of schistosomiasis: fever, eosinophilia, urticaria, and gastrointestinal disturbances. Moreover, five American POWs were subsequently diagnosed with it after being released, when they were found to be passing viable eggs of *S. japonicum* in their stool [9,13]. In addition, a high prevalence of active schistosomiasis was found among Puerto Rican nationals applying for enlistment into the U.S. Army, during WWII, when potential recruits were rejected based on positive stool examinations [9]. Schistosomiasis played a major role during the Chinese Civil War by stalling the Chinese Communist Party’s planned invasion of Taiwan [17]. At the time, schistosomiasis was a major public health problem in China, with approximately 32 million estimated cases of *S. japonicum* infections in the country in 1950 [17]. From late 1949 to early 1950, an epidemic of Katayama fever among the People’s Liberation Army troops which had trained weeks earlier in the waters of the Yangtze River Delta near Shanghai, known at the time to be endemic for schistosomiasis, likely delayed the invasion of Taiwan by several crucial months, during which time the Korean War began, and the U.S. began to provide significant military aid to Nationalist forces [17]. During the Korean war (1950–1953), schistosomiasis was less of an issue for the military, as schistosomiasis is not endemic to Korea [1,39]. Nevertheless, a retrospective study of death certificates and medical records aiming to determine the cause of death of POWs during the Korean War found one death due to schistosomiasis but concluded that the infected military personnel had likely moved from China or Japan [18]. During the Vietnam war, 69 American military personnel reportedly suffered from schistosomal dermatitis during operations in the Mekong Delta [19]. More recently, several countries reported clusters or outbreaks of schistosomiasis in troops deployed in endemic regions [20,21,22,23,24,25,26]. France reported an outbreak of *S. mansoni* infections having occurred in 1985 in 113 military personnel stationed in the Central African Republic, in the vicinity of a tributary of the Chari River [20]. All exposed soldiers were subjected on their return to France to a questionnaire, physical examination, stool examination and serological tests: 113 had positive serology, of which 89 also had positive stool examinations [20]. In 1995, 30 of 87 Brazilian service members who had participated in a peacekeeping mission in Mozambique had *S. haematobium* eggs in their urine and 50 had positive serology [22]. The infections were acquired while swimming in the Licungo River, Zambezia Province during leisure time [22]. In 2005–2006, 13 French soldiers contracted the disease while building bridges during operations in Ivory Coast [23]. All presented symptoms compatible with acute schistosomiasis (fever, rash, cough) and all were subsequently diagnosed by serological tests [23]. From September 2005 to December 2006, 49 out of a total of 280 Belgian soldiers deployed in Kalemie, Democratic Republic of Congo (DRC), along the west coast of Lake Tanganyika, were also infected [24]. The diagnoses were made after returning to Belgium by clinical and serological surveillance of all exposed personnel. Three of the forty-nine cases also presented *S. mansoni* ova in feces [24]. In June 2012, 107 of the 216 French soldiers exposed to the parasite while swimming in the M’Bari River (Central African Republic) were infected [25]. Examinations carried out after their return to France found blood hypereosinophilia in 87 patients, positive ELISA serology in 84, positive hemagglutination in 61, positive blood PCR in 57, positive stool PCR in 57, and *S. mansoni* ova in 3 on stool parasitological examination [25]. In the beginning of 2013, another sixteen out of sixty French personnel exposed during a mission to Madagascar were also diagnosed by positive serology, after one exposed soldier presented with hematospermia after returning home [25]. In Mars–April 2014, 7 of 28 U.S. service members who had freshwater exposure in the Nile River in Jinja, Uganda, were infected with *S. mansoni* [26]. The diagnosis was made by serological testing 12 weeks after the final exposure, after the unit’s return to the U.S. [26]. Other countries, such as Canada, Sweden, India, Germany, Portugal, and the U.S., also reported isolated cases of schistosomiasis in their returning personnel, especially after deployment to Africa [27,28,29,30,31,32].

### 3.3. Clinical Presentations in Non-Immune Hosts

Humans are infected when they come in contact with snail-infested freshwaters in endemic regions [1,2,4,9]. The snails act as intermediate host for the *Schistosoma* spp., and amplify their infective stages, called cercariae: the parasite undergoes asexual replication inside the snails, which eventually shed tens of thousands of cercariae into the water [4]. The cercariae penetrate the intact skin and transform into larvae called schistosomulae, then into juvenile worms which migrate to their respective end organs via the systemic circulation [1,4,9]. From a clinical point of view, schistosomiasis is divided into three stages: the first, called cercarial dermatitis, occurs 24 h after the penetration of the cercariae into the skin, the second, acute schistosomiasis, appears 3–8 weeks after infection, when symptomatic, and the chronic stage occurs months or years after infection and is a consequence of the formation of granulomas in the tissues around the schistosome eggs [5]. In endemic settings, children are often infected before they reach 2 years of age and the burden of infection increases in intensity during the next 10 years, as new exposures to cercariae-infested water occur and more worms colonize the child’s body [4]. While in endemic settings the most prevalent form of the disease is chronic schistosomiasis, which results from repeated exposure, the clinical presentation is different in travelers from non-endemic regions [2,4,5]. Military personnel deployed in endemic areas are a unique group of travelers, who are at high risk of schistosomiasis infections and reinfections through the nature of their job, which exposes them frequently to freshwater [8,9,10].

#### 3.3.1. Cercarial Dermatitis

Most cases of cercarial dermatitis, also called “swimmer’s itch”, are reported in infection-naïve travelers to schistosome-endemic regions who are exposed to schistosome antigens for the first time at an older age than usual in the local population [4,40]. An itchy maculopapular skin eruption, with 1 cm to 3 cm erythematous maculo-papules, may develop at the site of the percutaneous penetration by schistosomal cercariae [5,40]. The duration and severity of this reaction depend on the length of schistosomula stay in the dermis [40]. The lesions are most pronounced in infections with non-human *Schistosoma* spp., for which humans are an aberrant (dead-end) host, and whose schistosomulae cannot therefore migrate from the skin [40]. Usually, the disease is self-limiting within 1–3 weeks, and sometimes itching is the only symptom [5]. In this phase of infection, the serology is negative, and no eggs are found in urine or stool. Moreover, in case of infections with non-human *Schistosoma* spp., these tests will remain negative. A history of dermatitis limited to the body areas that were in contact with potentially contaminated freshwater is very suggestive of schistosomiasis, although other differential diagnoses are also possible [5]. The history of exposure to freshwater is essential to guide the diagnosis of cercarial dermatitis, so asking patients about activities such as bathing, crossing rivers or lakes, rafting, or even showering, is essential [7].

#### 3.3.2. Acute Schistosomiasis (Katayama Fever)

Acute schistosomiasis is most often found in non-immune travelers to schistosome-endemic regions [4,5]. It is sometimes referred to as Katayama fever or Katayama syndrome, and was first described in 1847 in Katayama district, Japan, where its etiologic agent was *S. japonicum* [5]. However, not all acute schistosomiasis patients present fever, and most cases nowadays are caused by *S. mansoni* or *S. haematobium* [5]. Acute schistosomiasis occurs weeks to two–three months after infection and is due to hypersensitivity reactions to worm maturation, egg production, release of egg antigen, and the host’s granulomatous and immune complex responses [4]. After penetrating the host through the intact skin, the cercariae metamorphose in larvae (schistosomulae) and develop an outer coating that resists a sustained host immune attack [38]. During this phase, the schistosomulae migrate to the pulmonary capillaries through the systemic circulation, become juvenile worms, and mature in the portal veins of the liver until reaching the adult form, leading to mating and egg production [4,38]. In this interval, called the prepatent period, the infection is ongoing, but eggs cannot be detected [4]. The production of eggs begins when the juvenile worms’ development into adult worms is complete, which occurs usually 4–8 weeks after skin penetration of the larvae, depending on the schistosome species [41].

Several pathophysiological hypotheses have been proposed for acute schistosomiasis [42]. Some consider that it is caused by the passage of soluble antigens from the eggs into the blood, giving rise to an inflammatory response that can be more or less severe, depending on the species [5], For other authors, it can occur in the absence of the eggs [41,42,43]. In a Dutch study, volunteers exposed to male-only *S. mansoni* parasites developed Katayama syndrome, despite the absence of eggs [43]. Symptoms usually appear between 3 and 8 weeks after exposure during the maturation of adult forms [41]. However, incubation periods of 1 to 12 weeks have been described [44]. In nonimmune travelers, the infection is symptomatic in 54–100% of cases [5]. The typical clinical presentation of acute schistosomiasis is a sudden onset of fever, malaise, myalgia, headache, eosinophilia, fatigue, urticaria, a nonproductive cough and abdominal pain lasting 2–10 weeks [4,5]. It can be accompanied by lymphadenopathy, hepatosplenomegaly, and diarrhea [9]. Some patients may develop persistent and more serious disease with weight loss, dyspnea, diffuse abdominal pain, toxemia, and widespread rash [5,40]. Between 50 and 75% of the cases have eosinophilia [5,41,44,45]. Sometimes, acute schistosomiasis can evolve rapidly to hepatic fibrosis, splenomegaly, and portal hypertension [40]. Wayward migration of the schistosomulae, especially through the central nervous system, may also cause significant morbidity and even death [9]. Neurological symptoms develop in approximately 2% of acute schistosomiasis cases [46]. They usually occur three weeks after the systemic symptoms, and may include headache (usually transient or intermittent), altered consciousness, coma, seizures, aphasia, blurred vision, cerebellar symptoms, cerebral infarcts, cerebral vasculitis, and acute encephalopathy [5,46]. The encephalopathy is more frequently caused by *S. japonicum* and *S. mansoni* [46]. Cases of spinal cord involvement, usually due to *S. mansoni*, have also been described [5]. Acute pulmonary involvement usually appears 3–8 weeks after penetration of the cercariae. The most frequent clinical manifestations are dyspnea, bronchospasm, dry cough, hemoptysis, chest pain, and wheezing [5]. The pulmonary symptoms can coincide with the fever of Katayama syndrome or may be independent and appear several weeks after the fever or continue after defervescence. The radiological involvement of acute pulmonary schistosomiasis is very variable, ranging between normal radiological exams and highly abnormal radiological pictures, independent of the clinical status of the patient (pathologic pulmonary imagery can be found in asymptomatic patients and vice versa) [5]. The most common radiological findings are poorly defined pulmonary nodules or interstitial pneumonitis like that seen in tropical pulmonary eosinophilia [5]. Cardiac involvement with chest pain, myocarditis, pericarditis or asymptomatic ischemia and ECG modifications such as T-wave and ST-segment abnormalities can also occur in acute schistosomiasis [5].

#### 3.3.3. Chronic Schistosomiasis

After maturing in the portal veins of the liver, the worms form couples and mate before migrating against the flow of the blood to the veinous plexuses of the target organs: the veins draining the main pelvic organs, including the bladder, uterus, and cervix for *S. haematobium* and the mesenteric venules for *S. mansoni*, *S. mekongi*, *S. intercalatum*, and *S. japonicum* [4,5,38,40]. The adult worm couples produce fertilized eggs, which are either shed into the environment with the feces or urine or are retained in host tissues where they induce inflammation and then die after 5–10 years [4,40]. It is the schistosome eggs, and not adult worms, that induce the morbidity caused by schistosome infections [4]. Many eggs are not excreted, and become permanently lodged in the intestines or liver (for *S. mansoni*, *S. japonicum*, *S. intercalatum* and *S. mekongi*) or in the bladder and urogenital system (for *S haematobium*), where they induce a granulomatous host immune response largely characterized by lymphocytes, eosinophils, and, alternatively, activated macrophages [4]. Inflammation stimulated by ova-secreted proteins and granuloma formation leads to primary tissue destruction and end organ damage. [9]. Secondary manifestations during this stage may also occur in the kidneys, lungs, central nervous system (CNS) or other ectopic sites [4,38]. Chronic morbidity is mainly related to the granulomatous reactions to the eggs, which are reversible in their early stage, but become irreversible once they progress to fibrosis [38,42]. Other pathophysiological mechanisms that lead to chronic morbidity are the calcification of the entrapped eggs, the deposition of schistosomal antigen–antibody complexes in the renal glomeruli, or the development of secondary amyloidosis [38]. Malignancy may complicate the chronic lesions in the urinary bladder or colon [4,40,47]. Chronic schistosomiasis is more frequently a pathology of populations living in endemic settings, who are repeatedly reinfected, than of non-immune travelers. However, there are reports of chronic schistosomiasis and urothelial cancer in veterans after deployment in endemic regions [31]. Noteworthy, is the fact that some experts argue that the concepts of acute versus chronic schistosomiasis should be abandoned in favor of the ‘reversible’ versus ‘irreversible’ designations, based on the presence or absence of reversible lesions (e.g., lung nodules, bladder polyps) or irreversible lesions (e.g., bladder cancer, periportal fibrosis), regardless of the presumed time from exposure [42].

- Urogenital Schistosomiasis:

More than 90% of schistosomiasis cases occur nowadays in Africa, and two-thirds of them are caused by *S. haematobium* [5,47]. *S. haematobium*, unlike the other human-infecting schistosome species, mainly migrates to the venous plexus surrounding the bladder, thus causing urinary schistosomiasis [1,4,5,29,47]. *S. haematobium* egg retention and granuloma formation in the urinary tract can cause terminal hematuria, increased frequency of micturition, dysuria, pyuria and hematospermia, bladder polyps, ulcers, and obstructive uropathies [4,5,27,38]. In the long term, it may cause chronic cystitis and ureterohydronephrosis, pyelonephritis, and renal failure from obstructive uropathy, as well as papillomas and bladder carcinoma [5,38]. *S. haematobium* genital involvement increases the risk factors of acquiring and spreading certain sexually transmitted diseases in tropical settings, such as the *human immunodeficiency virus* (HIV) and the human papillomavirus (HPV), by up to 30% [38]. Female genital manifestations may include hypertrophic and ulcerative lesions of the vulva, vagina, and cervix, and are associated with stress incontinence, decreased fertility, and spontaneous abortions [5]. Male genital schistosomiasis can involve the epididymis, spermatic cord, testes, and prostate gland, which can lead to sterility [38]. Genital lesions may be partially reversible, with treatment [38].

- Intestinal schistosomiasis:

Intestinal schistosomiasis is a very frequent complication, caused by infection with *S. mansoni*, *S. japonicum*, *S. intercalatum*, *S. mekongi*, and, occasionally, *S. haematobium* [5]. Its severity is higher in the cases of recurrent exposure, as it is related to the number of parasite eggs present in the mucosa of the intestinal tract, mainly the colon and rectum [5]. The most common symptoms are nonspecific, and include chronic or intermittent abdominal pain, asthenia, weight loss, anorexia, diarrhea, and anemia due to bleeding from ulcerations in the colon and rectum, which can mimic inflammatory bowel diseases [5]. In some patients, granulomatous inflammation will degenerate into polyps, which is the most common intestinal lesion in chronic intestinal schistosomiasis [33]. All parts of the colon can be affected, but, most frequently, the descending colon or rectum is involved [33].

- Hepatosplenic schistosomiasis:

Hepatosplenic schistosomiasis is a heterogeneous condition, ranging from a mildly symptomatic to life-threatening disease [4,5]. Several studies have demonstrated a correlation between disease severity and quantitative fecal egg output [38]. The most common presentation of hepatosplenic schistosomiasis is upper gastrointestinal bleeding, leading to severe anemia [5]. Periportal fibrosis, also called “*Symmers’ pipestem fibrosis*”, is the most common complication [38]. The fibrosis leads to portal hypertension and gastrointestinal bleeding. In contrast to cirrhosis, in hepatosplenic schistosomiasis, hepatic function is preserved overall, except in the presence of other infections such as chronic hepatitis B or C [38].

- Neuroschistosomiasis:

Neuroschistosomiasis is the most feared clinical syndrome associated with *Schistosoma* spp. infections, as it could occur even with low egg load. It can encompass a large range of signs and symptoms, such as increased intracranial pressure, myelopathy, and radiculopathy [38,40]. The lesions can evolve to irreversible glial scars if left untreated [40]. Ectopic eggs can gain access to the CNS through the anastomosis in between the lumbar veins, which are tributaries of the inferior vena cava, and the internal vertebral venous plexus [5,38,40]. Ectopic eggs may be deposited and provoke granuloma formation in the adjacent spinal cord or may be pushed towards the brain during coughing or straining [40]. *S. japonicum* is more commonly encountered in cerebral schistosomiasis, because the small size of its eggs facilitates their journey towards the brain, including the cortex, subcortical white matter, basal ganglia, and internal capsule, while *S. mansoni* and *S. haematobium* are more commonly reported in myelopathy of the lumbosacral region, for anatomical reasons [40]. Complications of cerebral disease include headache, visual impairment, delirium, seizures, motor deficits, and ataxia. Spinal involvement may include lumbar pain, lower limb radicular pain, muscle weakness, sensory loss, bladder dysfunction, and transverse myelitis [38,40].

- Cardiopulmonary schistosomiasis:

Cardiopulmonary disease occurs due to egg embolization, granuloma formation and immune-mediated endothelial proliferation in both pre-and post-alveolar capillaries, resulting in obstructive pulmonary hypertension [40]. This is usually asymptomatic, but may be associated with shortness of breath. Right ventricular failure, with severe tricuspid incompetence, may occur in terminal phases [40].

- Malignancy:

Oncologic complications of schistosomiasis are a public health problem in regions endemic for *S. haematobium* [47]. Chronic *S. haematobium* infection has been associated with the development of squamous cell carcinoma and urothelial carcinoma of the urinary bladder [47]. The continuous inflammatory reaction to the eggs leads to parenchymal tissue destruction, inflammation, fibrosis, granulomas, and fibrotic nodules named sandy patches, and may ultimately cause carcinogenesis [47]. While the carcinogenic role of the other *Schistosoma* spp. is not as clear as that of *S. haematobium*, the association of hepatosplenic *S. mansoni* infections with hepatitis B or C viral infections is known to increase the risk of liver malignancy [47].

### 3.4. Diagnosis

The diagnosis of schistosomiasis depends on its infection stage and intensity [2,4]. Several tests are available for clinical practice, but all have limitations in specificity and sensitivity. The diagnostic gold standard for active schistosomiasis is the detection of viable eggs in urine (*S. haematobium*), feces (*S. japonicum*, *S. mansoni and other schistosomes with intestinal tropism*), or tissue biopsies [2,4]. However, this poses several hurdles when diagnosing particular groups, such as military personnel. Firstly, during the early stages of the disease, no eggs are excreted, and the serological tests are still negative [2,4]. Therefore, a thorough interrogation of the patients about history of exposure to freshwater is essential, to guide the diagnosis in the acute stages [7]. During acute schistosomiasis, only 50–75% of cases have eosinophilia, so its absence does not exclude the diagnosis of acute schistosomiasis, and requires repeating the laboratory tests after 2–3 weeks [5,41,45]. There is no consensus about the cut-off of eosinophil count which would raise suspicion in travelers [41]. The onset of eosinophilia is often delayed by several days, as compared to the clinical syndrome. One study in travelers showed that eosinophilia usually appears late, 21 days after the fever and up to 47 days (range 25–119) after the exposure [48]. In the study conducted by Aerssens, in Belgian soldiers returning from the DRC, the eosinophil counts were not sensitive enough to suspect or to rule out infection in most patients who were asymptomatic or who had only minor symptoms at the time of diagnosis [24]. Acute schistosomiasis may also present other nonspecific biological findings such as anemia, elevated IgE levels, altered transaminases, hypoalbuminemia, and hypergammaglobulinemia, further complicating the differential diagnosis [5]. When the CNS is involved, cerebrospinal fluid examination may be normal or show nonspecific findings [5]. Another obstacle in diagnosing infected military personnel during deployment in resource-limited settings is that diagnostic options are usually scarce, so diagnosis is frequently based on epidemiological information obtained during cross-sectional or post-deployment assessments [30]. Even after returning from deployment, laboratory diagnosis of schistosomiasis in patients with symptoms compatible with Katayama syndrome can be particularly difficult, due to the poor sensitivity of common diagnostic tests during early infection, such as serology and microscopy [49]. Even during later stages, when eggs are excreted, microscopy lacks sensitivity and requires examining multiple consecutive samples, particularly when the worm burden is low, which is mostly the case in travelers or deployed military personnel, [50,51]. Molecular biology techniques such as real-time polymerase chain reactions (PCRs) for the detection of *Schistosoma* spp. in serum have shown promising results for early diagnosis of acute schistosomiasis in nonimmune travelers, in military as in civilian settings [25,51,52,53]. Because egg loads are usually low in nonimmune travelers, diagnosis in this group is routinely performed by serological tests after returning from the endemic setting [52]. The study conducted by Aerssens et al. in 197 exposed Belgian military personnel after deployment to a Schistosoma-endemic region in the DRC, in which 61% of the 49 seropositive cases were asymptomatic after a 768-day median time from exposure to diagnosis, emphasized the need for active systematic post-tropical screening in military personnel [24]. However, the sensitivity of conventional serology is low in acute schistosomiasis. For example, in the study conducted by Soentjens et al. in 2016, more than one-third of a cluster of returning travelers from Mali did not have schistosome antibodies at initial presentation [52]. Another retrospective study, conducted by Vanbrabant et al. from 1 January 2018 to 1 January 2019, on a cohort of 946 Belgian personnel returning mainly from Sub-Saharan Africa, also showed that the attack rates and incidence rates as diagnosed by serology are low [34]. Some aspects to be considered are the fact that serological cross-reactions between helminths, especially trematodes, are a well-known phenomenon, and that low pre-test probabilities in low-prevalence populations such as military personnel from non-endemic regions may result in false positive results [34]. Also, the semi-quantitative serological results are not related to the worm burden, and the tests may remain positive for several years, even after successful treatment, so serological methods are not useful for distinguishing between active infections and past infections, and in post-treatment monitoring [50,54]. Active infections, where viable worms are present in the bloodstream, can be detected by the quantification of genus-specific antigens originating from the gut of the adult worm in the host circulatory system [55]. One of these antigens, the circulating anodic antigen (CAA), can be detected in serum and urine through a highly specific and ultra-sensitive lateral flow (LF) test that applies up-converting reporter particles (UCPs) [56,57]. CAA concentrations decline rapidly following successful treatment, making the UCP-LF CAA assay highly suitable to monitor a cure [43]. However, CAA is yet to be implemented in the routine diagnosis and follow-up of schistosomiasis in travelers or deployed military personnel.

### 3.5. Treatment

Helminths are relatively cheap and easy to treat, if diagnosed early [7]. Praziquantel (PZQ), a pyrazino–isoquinoline derivative developed by Bayer in the 1970s, is still the drug of choice for schistosomiasis [1,4,5,47]. It is effective against all *Schistosoma* spp., but for full efficacy it needs an effective host antibody response [4]. PZQ acts against adult worms of all *Schistosoma* spp., but has poor activity against juvenile worms [4,5,42]. It is simple to administer, safe, well-tolerated, and cheap [4,58]. The World Health Organization (WHO)-recommended, standard-care, 40 mg/kg single-dose PZQ treatment reaches an aggregated parasitological cure rate of 75% for both predominant species, *S. haematobium* and *S. mansoni* [58]. For *S. japonicum* and *S. mekongi*, the recommended dose is 60 mg/kg [4,40]. Common side-effects are abdominal pain, transient nausea, headache, dizziness, rash, pruritus, and transient passage of blood in stool [4,59]. High-burden infections correlate with higher risk of side-effects, which peak about 2–4 h after drug intake and are self-limited [4]. PZQ can be used safely in pregnancy after the first trimester [4]. It is absorbed well, but undergoes extensive first-pass hepatic clearance, it is secreted in breast milk and is metabolized by the liver, and its metabolites, which are inactive, are excreted in the urine [60]. Active lesions respond rapidly to antischistosomal chemotherapy, but PZQ cannot be used for chemoprophylaxis or for the treatment of acute schistosomiasis, since it is active only against mature worms [40]. Artemisinin derivates are promising new agents that can effectively kill the invading cercariae, maturing schistosomulae and the mature adult worms, by interfering with the parasite’s glycolytic pathways [40]. In a recent trial conducted in schoolchildren from Northern Senegal, a 3-day course of artesunate–mefloquine at antimalarial dosage was noninferior to a standard single dose of praziquantel, for the treatment of schistosomiasis, mainly *S. haematobium* infections [58]. Moreover, both drugs also have an effect on juvenile worms, in contrast to PZQ [58]. Corticosteroids, such as prednisone 1.5–2.0 mg/kg per day for three weeks, are used to treat Katayama syndrome, as they help diminish the inflammatory reaction [38,42]. Corticosteroids are also used for the treatment of schistosomal encephalopathy during the egg-laying stage and, may be needed as adjuvants to PZQ and anticonvulsants in neuroschistosomiasis [60].

### 3.6. Prevention in the Military Setting

The prevention and control of schistosomiasis at a public health level requires a multi-faceted strategy, with several complementary and integrated One Health approaches [1,59]. Effective treatment of people, so that their excreta do not contain eggs, the prevention of sewage contamination of freshwater, the elimination of intermediate host snails, and the prevention of human contact with water containing infected snails are all interventions that can help to prevent transmission [4,35,36,39]. In the military setting, as deployments in tropical conditions usually include intensive soil and freshwater contact, the risk of being infected with helminths is higher, compared to that of a regular traveler [8]. Therefore, these high-risk groups should be tested prior to and after deployment and, if seropositive, should be treated with praziquantel. Education is one of the cornerstones of prevention. For example, the whole program of control of schistosomiasis in the U.S. military during WWII was a preventive one, with a great emphasis on education of the at-risk troops [16]. Good schistosomiasis discipline on the part of troops demands an intensive educational program, through which the personnel become adequately informed as to the dangers of exposure to fresh water in endemic areas [16]. Unit commanders and medical personnel should provide pre-deployment health protection briefs in order to educate the personnel about the risks of exposure to freshwater and the available risk-mitigation strategies [28,40]. A good knowledge of the endemic areas of schistosomiasis and their endemic hosts is also paramount. Unit commanders and medical personnel should discourage unnecessary fresh water contact in endemic regions [26]. Cercariae can remain infective in freshwater for 1–3 days, and environmental changes can both increase and decrease transmission [4]. Topical lotions of N, N-diethyl-m-toluamide (DEET) have been shown to have some effect against cercariae [61], but further studies showed no conclusive evidence of prevention against *Schistosoma* infection. If freshwater exposure does occur in endemic areas, the personnel should be tested 6 to 8 weeks after exposure, and treated if necessary [26]. It is important to know that a previous infection, and therefore the presence of antibodies against *Schistosoma*, is not protective against reinfections. Also, PZQ is effective against adult worms, but not against immature or developing worms [4,5,42].

## 4. Future Directions

Due to funding issues and the complex immunological pathway involvement in schistosomal infections, researchers in the field of vaccinology have struggled for many years [62]. No licensed/commercial schistosomiasis vaccine is currently available on the market against any *Schistosoma* parasite, but there have been some promising results in clinical trials, and currently there are four major leading human-based schistosomiasis vaccines that are undergoing clinical trials at various development phases: Sm-p80, Sm-TSP-2, Sh28GST, and Sm-14 [59].

## 5. Conclusions

Given the sustained military presence in endemic regions, schistosomiasis remains a major problem for the military. Not only can it pose problems to the personnel during deployment to endemic areas, but, as infections can be asymptomatic or misdiagnosed, they can persist for several years, even decades, and, if undiagnosed can have devastating implications later in life.

## Figures and Tables

**Table 1:** Inclusion and exclusion criteria to identify articles suitable for full review.

Inclusion Criteria	Exclusion Criteria
Original studies	Editorials
Review articles	Letters to the Editor
Addressing schistosomiasis in military personnel	Correspondences
	Animal studies Full text not in English or French

## References

[1] WHO (2023). Fact Sheet Schistosomiasis. https://www.who.int/news-room/fact-sheets/detail/schistosomiasis.

[2] Clerinx J., Van Gompel A. (2011). Schistosomiasis in travellers and migrants. Travel. Med. Infect. Dis..

[3] Mohamed I., Kinung’hi S., Mwinzi P.N., Onkanga I.O., Andiego K., Muchiri G., Odiere M.R., Vennervald B.J., Olsen A. (2018). Diet and hygiene practices influence morbidity in schoolchildren living in Schistosomiasis endemic areas along Lake Victoria in Kenya and Tanzania—A cross-sectional study. PLoS Negl. Trop. Dis..

[4] Colley D.G., Bustinduy A.L., Secor W.E., King C.H. (2014). Human schistosomiasis. Lancet.

[5] Carbonell C., Rodríguez-Alonso B., López-Bernús A., Almeida H., Galindo-Pérez I., Velasco-Tirado V., Marcos M., Pardo-Lledías J., Belhassen-García M. (2021). Clinical Spectrum of Schistosomiasis: An Update. J. Clin. Med..

[6] Soonawala D., van Lieshout L., den Boer M.A., Claas E.C., Verweij J.J., Godkewitsch A., Ratering M., Visser L.G. (2014). Post-travel screening of asymptomatic long-term travelers to the tropics for intestinal parasites using molecular diagnostics. Am. J. Trop. Med. Hyg..

[7] Agbessi C.A., Bourvis N., Fromentin M., Jaspard M., Teboul F., Bougnoux M.E., Hanslik T. (2006). La bilharziose d’importation chez les voyageurs: Enquête en France métropolitaine [Acute schistosomiasis in French travellers]. Rev. Med. Interne.

[8] Lindrose A.R., Mitra I., Fraser J., Mitre E., Hickey P.W. (2021). Helminth infections in the US military: From strongyloidiasis to schistosomiasis. J. Travel. Med..

[9] Crum N.F., Aronson N.E., Lederman E.R., Rusnak J.M., Cross J.H. (2005). History of U.S. military contributions to the study of parasitic diseases. Mil. Med..

[10] Strohmayer J., Matthews I., Locke R. (2016). Schistosomiasis: Traverers in Africa. J. Spec. Oper. Med..

[11] Reeves W.K., Bettano A.L. (2014). A review of mortality from parasitic and vector-borne diseases in the U.S. Air Force from 1970 to 2012. J. Parasitol..

[12] Page M.J., McKenzie J.E., Bossuyt P.M., Boutron I., Hoffmann T.C., Mulrow C.D., Shamseer L., Tetzlaff J.M., Akl E.A., Brennan S.E. (2021). The PRISMA 2020 statement: An updated guideline for reporting systematic reviews. BMJ.

[13] Preventive Medicine in WWII, Volume 5 Communicable Diseases—Chapter 6: Schiostosomiasis. https://achh.army.mil/history/book-wwii-communicablediseasesv5-chapter6.

[14] Guly H. (2016). Edward Leicester Atkinson (1881–1929): Antarctic explorer, scientist and naval surgeon. J. Med. Biogr..

[15] Stothard J.R., Kabatereine N.B., Archer J., Al-Shehri H., Tchuem-Tchuenté L.A., Gyapong M., Bustinduy A.L. (2017). A centenary of Robert T. Leiper’s lasting legacy on schistosomiasis and a COUNTDOWN on control of neglected tropical diseases. Parasitology.

[16] Leiper R.T. (1918). Report on the results of the bilharzia mission in Egypt, 1915. J. R. Army Med. Corps.

[17] Turner M.D., Sapp J. (2024). Schistosomiasis: The Disease That Saved Taiwan. Mil. Med..

[18] Huh S. (2014). Parasitic diseases as the cause of death of prisoners of war during the Korean War (1950–1953). Korean J. Parasitol..

[19] Allen A.M., Taplin D., Legters L.J., Ferguson J.A. (1974). Letter: Schistosomes in Vietnam. Lancet.

[20] Gras C., Martet G., Renoux E., Lecamus J.L., Aubry P. (1987). Une épidémie de bilharziose à schistosoma mansoni. 113 observations dans une collectivité militaire au retour d’Afrique centrale [An outbreak of Schistosoma mansoni bilharziasis. 113 cases in a military unit returning from Central Africa]. Rev. Med. Interne.

[21] Rocha M.O., Pedroso E.R., Neves J., Rocha R.S., Greco D.B., Lambertucci J.R., Rocha R.L., Katz N. (1993). Characterization of the non-apparent clinical form in the initial phase of schistosomiasis mansoni. Rev. Inst. Med. Trop. Sao Paulo..

[22] da Silva I.M., Tsang V., Noh J., Rey L., Conceição M.J. (2006). Clinical and laboratorial evaluation of urinary schistosomiasis in Brazilians after staying in Mozambique. Rev. Soc. Bras. Med. Trop..

[23] Haus-Cheymol R., Burlation G., Berger F., Wendling G., Schwartzbrod P.E., Cardona F., Terrier F., Spiegel A. (2007). Clustered cases of urinary and intestinal bilharziasis in French military personnel. Med. Trop..

[24] Aerssens C., De Vos D., Pirnay J.P., Yansouni C., Clerinx J., Van Gompel A., Soentjens P. (2011). Schistosomiasis in Belgian military personnel returning from the Democratic Republic of Congo. Mil. Med..

[25] Biance-Valero E., De Laval F., Delerue M., Savini H., Cheinin S., Leroy P., Soullié B. (2013). Épidémies de bilharziose chez des militaires projetés en zone d’endémie, techniques de diagnostic classique et mise au point de techniques de réaction en chaîne de la polymérase (PCR) en temps réel [Epidemics of schistosomiasis in military staff assigned to endemic areas: Standard diagnostic techniques and the development of real-time PCR techniques]. Med. Sante Trop..

[26] Maluil S., Stevens R.A. (2016). Clinical Report: Schistosomiasis Exposure in U.S. Service Personnel During Whitewater Rafting on the Nile River in Jinja, Uganda. Mil. Med..

[27] Vail W.J. (1958). Urinary schistosomiasis: Report of a case in a Canadian soldier. Can. Med. Assoc. J..

[28] Bengtsson E., Pellegrino J. (1966). Bilharziasis in Swedish military personnel returning from the Democratic Republic of the Congo. Bull. World Health Organ..

[29] Ashta K.K., Kumar R. (2010). A Case of Katayama Fever (Acute Schistosomiasis). Med. J. Armed Forces India.

[30] Schawaller M., Wiemer D., Hagen R.M., Frickmann H. (2023). Infectious diseases in German military personnel after predominantly tropical deployments: A retrospective assessment over 13 years. BMJ Mil. Health..

[31] Vieira P., Miranda H.P., Cerqueira M., Delgado Mde L., Coelho H., Antunes D., Cross J.H., da Costa J.M. (2007). Latent schistosomiasis in Portuguese soldiers. Mil. Med..

[32] Itani Y., Jones E., Kachur M., Hughey S., Nella V., Miller J. (2024). Gross haematuria at sea: Schistosomiasis in a US Military Servicemember. BMJ Mil. Health.

[33] Al-Zubaidi A.M., Bashanfer G.A., Alqannas M.H., Al Atawi A.S. (2020). Colonic Polyps an Unusual Manifestation of Schistosomiasis. Am. J. Case Rep..

[34] Vanbrabant P., Damanet B., Maussen C., Van Esbroeck M., Soentjens P. (2021). Screening the asymptomatic soldiers after a stay in sub-Saharan Africa. A retrospective observational study. Travel. Med. Infect. Dis..

[35] Nelwan M.L. (2019). Schistosomiasis: Life Cycle, Diagnosis, and Control. Curr. Ther. Res. Clin. Exp..

[36] Gurarie D., Lo N.C., Ndeffo-Mbah M.L., Durham D.P., King C.H. (2018). The human-snail transmission environment shapes long term schistosomiasis control outcomes: Implications for improving the accuracy of predictive modeling. PLoS Negl. Trop. Dis..

[37] Noël H., Ruello M., Maccary A., Pelat C., Sommen C., Boissier J., Barré-Cardi H., Fillaux J., Termignon J.L., Debruyne M. (2018). Large outbreak of urogenital schistosomiasis acquired in Southern Corsica, France: Monitoring early signs of endemicization?. Clin. Microbiol. Infect..

[38] Verjee M.A. (2019). Schistosomiasis: Still a Cause of Significant Morbidity and Mortality. Res. Rep. Trop. Med..

[39] Sandbach F.R. (1976). The history of schistosomiasis research and policy for its control. Med. Hist..

[40] Barsoum R.S., Esmat G., El-Baz T. (2013). Human schistosomiasis: Clinical perspective: Review. J. Adv. Res..

[41] Jauréguiberry S., Paris L., Caumes E. (2010). Acute schistosomiasis, a diagnostic and therapeutic challenge. Clin. Microbiol. Infect..

[42] Gobbi F., Tamarozzi F., Buonfrate D., van Lieshout L., Bisoffi Z., Bottieau E. (2020). TropNet Schisto Task Force. New Insights on Acute and Chronic Schistosomiasis: Do We Need a Redefinition?. Trends Parasitol..

[43] Langenberg M.C.C., Hoogerwerf M.A., Koopman J.P.R., Janse J.J., Kos-van Oosterhoud J., Feijt C., Jochems S.P., de Dood C.J., van Schuijlenburg R., Ozir-Fazalalikhan A. (2020). A controlled human Schistosoma mansoni infection model to advance novel drugs, vaccines and diagnostics. Nat. Med..

[44] Bottieau E., Clerinx J., de Vega M.R., den Enden E.V., Colebunders R., Van Esbroeck M., Vervoort T., Van Gompel A., Ende J.V.D. (2006). Imported Katayama fever: Clinical and biological features at presentation and during treatment. J. Infect..

[45] Ross A.G., Vickers D., Olds G.R., Shah S.M., McManus D.P. (2007). Katayama syndrome. Lancet Infect. Dis..

[46] Jauréguiberry S., Caumes E. (2008). Neurological involvement during Katayama syndrome. Lancet Infect. Dis..

[47] Santos L.L., Santos J., Gouveia M.J., Bernardo C., Lopes C., Rinaldi G., Brindley P.J., Costa J.M.C.D. (2021). Urogenital Schistosomiasis-History, Pathogenesis, and Bladder Cancer. J. Clin. Med..

[48] Leshem E., Meltzer E., Marva E., Schwartz E. (2009). Travel-related Schistosomiasis Acquired in Laos. Emerg. Infect. Dis..

[49] Tamarozzi F., Mazzi C., Antinori S., Arsuaga M., Becker S.L., Bottieau E., Camprubi-Ferrer D., Caumes E., Duvignaud A., Grobusch M.P. (2024). Consensus definitions in imported human schistosomiasis: A GeoSentinel and TropNet Delphi study. Lancet Infect. Dis..

[50] Weerakoon K.G., Gobert G.N., Cai P., McManus D.P. (2015). Advances in the diagnosis of human schistosomiasis. Clin. Microbiol. Rev..

[51] Soentjens P., Cnops L., Huyse T., Yansouni C., De Vos D., Bottieau E., Clerinx J., Van Esbroeck M. (2016). Diagnosis and Clinical Management of Schistosoma haematobium-Schistosoma bovis Hybrid Infection in a Cluster of Travelers Returning from Mali. Clin. Infect. Dis..

[52] Cnops L., Tannich E., Polman K., Clerinx J., Van Esbroeck M. (2012). Schistosoma real-time PCR as diagnostic tool for international travellers and migrants. Trop. Med. Int. Health.

[53] Cnops L., Soentjens P., Clerinx J., Van Esbroeck M. (2013). A Schistosoma haematobium- specific real-time PCR for diagnosis of urogenital schistosomiasis in serum samples of international travelers and migrants. PLoS Negl. Trop. Dis..

[54] de Jonge N., Polderman A.M., Hilberath G.W., Krijger F.W., Deelder A.M. (1990). Immunodiagnosis of schistosomiasis patients in The Netherlands: Comparison of antibody and antigen detection before and after chemotherapy. Trop. Med. Parasitol..

[55] van Lieshout L., Polderman A.M., Deelder A.M. (2000). Immunodiagnosis of schistosomiasis by determination of the circulating antigens CAA and CCA, in particular in individuals with recent or light infections. Acta Trop..

[56] van Dam G.J., de Dood C.J., Lewis M., Deelder A.M., van Lieshout L., Tanke H.J., van Rooyen L.H., Corstjens P.L. (2013). A robust dry reagent lateral flow assay for diagnosis of active schistosomiasis by detection of Schistosoma circulating anodic antigen. Exp. Parasitol..

[57] Corstjens P.L., De Dood C.J., Kornelis D., Fat E.M., Wilson R.A., Kariuki T.M., Nyakundi R.K., Loverde P.T., Abrams W.R., Tanke H.J. (2014). Tools for diagnosis, monitoring and screening of Schistosoma infections utilizing lateral-flow based assays and upconverting phosphor labels. Parasitology.

[58] Bottieau E., Mbow M., Brosius I., Roucher C., Gueye C.T., Mbodj O.T., Faye B.T., De Hondt A., Smekens B., Arango D. (2024). Antimalarial artesunate-mefloquine versus praziquantel in African children with schistosomiasis: An open-label, randomized controlled trial. Nat. Med..

[59] Siddiqui A.J., Bhardwaj J., Saxena J., Jahan S., Snoussi M., Bardakci F., Badraoui R., Adnan M. (2023). A Critical Review on Human Malaria and Schistosomiasis Vaccines: Current State, Recent Advancements, and Developments. Vaccines.

[60] Gray D.J., Ross A.G., Li Y.S., McManus D.P. (2011). Diagnosis and management of schistosomiasis. BMJ.

[61] Ramaswamy K., He Y.X., Salafsky B., Shibuya T. (2003). Topical application of DEET for schistosomiasis. Trends Parasitol..

[62] Ogongo P., Nyakundi R.K., Chege G.K., Ochola L. (2022). The road to elimination: Current state of schistosomiasis research and progress towards the end game. Front. Immunol..

